# Acid Dyspepsia

**Published:** 1905-09-09

**Authors:** 


### ACID DYSPEPSIA.

Dr. Robert Hutchison,[1] in a clinical lecture on this disease, observes that it is essentially a disease of the male. It is, as its name implies, a dyspepsia marked by an excessive secretion of hydrochloric acid by the stomach, being also known by the term "hypersthenic dyspepsia" in contradistinction from the "asthenic dyspepsia" commonly seen among women. The beginning of the disease is usually as follows: The patient at the period of adolescence has been subject to intermittent attacks of "indigestion," whose characteristic feature is pain. This pain is peculiar in that it is not observed for some time after the ingestion of food, and reaches its maximum severity some two hours or more after a meal. Also the pain is relieved by eating, for the time at all events. To this stage of acid dyspepsia the term "hyperchlorhydria" is applied, to indicate that the anomaly is an (intermittent) over-production of hydrochloric acid. The author observes that the usually-accepted pathology of the condition probably does not cover the whole ground, because the healthy stomach is not so sensitive to hydrochloric acid as the theory would imply. He himself, at a time when he was suffering from such hyperchlorhydria, took some ·6 per cent. hydrochloric acid without causing any pain at all. He therefore concludes that hyperæsthesia of the stomach is an element of the disease. This sounds a reasonable proposition, *per se*, but we fail to comprehend why, in the experiment abovementioned, artificially-introduced hydrochloric acid should have failed to affect an admittedly susceptible and presumably hyperæsthetic mucous membrane, if the importance of hydrochloric acid be as great as Dr. Hutchison believes. The next stage in the process is a phase of permanent hypersecretion. This is evidenced by the fact that the passage of a stomach-tube upon patients so affected will show that even in the morning, that is to say, before any food has been taken, the stomach contains acid gastric juice. This permanent presence of acid secretion in the stomach is succeeded by a condition of spasm on the part of the pyloric sphincter leading to cramp-like epigastric pains. The final phase of acid dyspepsia is dilatation and hypertrophy of the stomach. This is the only stage at which physical signs of the lesion can present themselves; they consist of an enlargement of the stomach resonance and an audible splash of the contents on suitable manipulation.

This hypersecretion appears to be a pure neurosis, and since we know no drug which is capable of controlling the secretion, treatment of the early stages resolves itself into the administration of drugs which will neutralise it. These are the alkaline carbonates, especially those of the earthy metals. The advantage of these over salts of the alkaline metals lies in the fact that any excess of the former remains undissolved in the stomach, while the latter, when present in excess, being soluble, merely provokes an increased acid secretion. Dr. Hutchison advises that carbonate of magnesium and carbonate of bismuth, of each 15 grains, be prescribed in cachets, one or more to be taken as soon as the epigastric pain appears. He does not consider that diet is a matter of much importance at this stage.

When the acid dyspepsia is more permanent it is advised that the patient be put to bed, that hot fomentations be applied to the epigastrium continuously for at least a week, that the diet should be restricted to milk, as being a good neutraliser of acid, and that neutralising powders be given as above described. The effect of the fomentations is to diminish the hyperæsthesia of the stomach, and the associated treatment tends to allay the spasm of the pylorus.

In the final stage, if the treatment above recommended be unattended by success, it is likely that the pylorus is permanently thickened or actually narrowed by the after-effects of ulceration. If this be the case, the advisability of gastro-enterostomy must be considered, for it is in cases of this kind that the operation is likely to yield its most brilliant results.

[1] Clinical Jour., Aug. 16, 1905.

